# A Case of Adult Attention Deficit Hyperactivity Disorder with Non-Organic Psychosis Comorbidity

**DOI:** 10.1192/j.eurpsy.2022.1190

**Published:** 2022-09-01

**Authors:** S. Akyıldırım Çor, M. Duman, Ö. Uzun

**Affiliations:** Gülhane Training and Research Hospital, Department Of Psychiatry, Ankara, Turkey

**Keywords:** Psychosis, Attention Deficit, hyperactivity, comorbidity

## Abstract

**Introduction:**

Although ADHD is the most frequently diagnosed psychiatric disorder in childhood, the majority of adults with ADHD are not diagnosed and 90% of the cases remain untreated. One of the main reasons that may lead to the missed diagnosis of ADHD in adults may be the high rate of comorbid psychiatric conditions masking the main symptoms.

**Objectives:**

In this study, it was aimed to present a case who was followed up with the diagnosis of ADHD since childhood and developed psychosis after a recent traumatic life event.

**Methods:**

A 19-year-old male patient was consulted because of his complaints of persecution delusions, and disorganized speech that started 2 years ago. IIt was learned that the first psychiatry application of the patient was 10 years ago with complaints of impulsivity, aggression, increased psychomotor movements, and methylphenidate treatment was started during this period. The patient, whose current clinical picture was evaluated as psychosis, was discharged after the symptoms subsided with paliperidone depot 100mg/month treatment after hospitalization. It is understood that his psychotic complaints completely regressed in the follow-ups.

**Results:**

It is stated that approximately 80% of adult ADHD cases have at least one accompanying psychiatric disorder. However, there are limited studies in the literature on the relationship between psychotic disorders and ADHD.

**Conclusions:**

Recent studies indicate that beyond the fact that ADHD is a feature of the schizophrenia prodrome, ADHD diagnosis may be associated with an increased risk of psychosis in the future. Therefore, this association can be better clarified in further studies on comorbidities.

**Disclosure:**

No significant relationships.

